# Bioinformatics for Next Generation Sequencing Data

**DOI:** 10.3390/genes1020294

**Published:** 2010-09-14

**Authors:** Alberto Magi, Matteo Benelli, Alessia Gozzini, Francesca Girolami, Francesca Torricelli, Maria Luisa Brandi

**Affiliations:** 1 Diagnostic Genetic Unit, Careggi Hospital, Azienda Ospedaliera Universitaria Careggi, University of Florence, Florence, Italy; E-Mails: matteo.benelli@gmail.com (M.B.); gozzinial@aou-careggi.toscana.it (A.G.); girolamif@aou-careggi.toscana.it (F.G.); torricellif@aou-careggi.toscana.it (F.T.); 2 Center for the Study of Complex Dynamics, University of Florence, Florence, Italy; 3 Surgical Critical Care, University of Florence, Florence, Italy; 4 INFN, Sezione di Firenze, Firenze, Italy; 5 Department of Internal Medicine, University of Florence Medical School, Florence, Italy; E-Mail: m.brandi@dmi.unifi.it; † These authors contributed equally to this work

**Keywords:** sequencing, data analysis, bioinformatics

## Abstract

The emergence of next-generation sequencing (NGS) platforms imposes increasing demands on statistical methods and bioinformatic tools for the analysis and the management of the huge amounts of data generated by these technologies. Even at the early stages of their commercial availability, a large number of softwares already exist for analyzing NGS data. These tools can be fit into many general categories including alignment of sequence reads to a reference, base-calling and/or polymorphism detection, *de novo* assembly from paired or unpaired reads, structural variant detection and genome browsing. This manuscript aims to guide readers in the choice of the available computational tools that can be used to face the several steps of the data analysis workflow.

## 1. Introduction 

The last few years have seen the emergence of several high-throughput sequencing (HTS) (or Next-Generation Sequencing, NGS) platforms that are based on various implementations of cyclic-array sequencing. The concept of cyclic-array sequencing can be summarized as the sequencing of a dense array of DNA features by iterative cycles of enzymatic manipulation and imaging-based data collection [1]. The commercial products that are based on this sequencing technology include Roche’s 454, Illumina’s Genome Analyzer, ABI’s SOLiD and the Heliscope from Helicos. Although these platforms are quite diverse in sequencing biochemistry as well as in how the array is generated, their work flows are conceptually very similar. All of them allow the sequencing of millions of short sequences (reads) simultaneously, and are capable of sequencing a full human genome per week at a cost 200-fold less than previous methods. Moreover, HTS platforms allow the generation of many kinds of sequence data: for example, they are used to make *de novo* sequencing, to resequence individuals when a reference genome already exists, sequence RNA to quantify expression level (RNA-seq) [2,3,4] and study the regulation of genes by sequencing chromatin immunoprecipitation products (ChIP-Seq) [5]. The advent of HTS platforms has opened many opportunities for genomic variant discovery [6,7,8]. Although the bioinformatics community has solved many aspects of the analysis of all of these kinds of data, here we will focus our attention only on the algorithms that have been developed for the discovery of genomic variants. 

In the following sections of this review we will describe the HTS technologies and the data generated by them, and then we will focus on the statistical methods and algorithms used for the detection of genomic variants.

## 2. High Throughput Sequencing Technologies

The workflows of all of the currently available HTS technologies are very similar. The first step of the sequencing process consists of genomic DNA fragmentation and ligation to common adaptors. In this first step, all of the HTS technologies are able to use alternative protocols in order to generate jumping libraries of mate-paired tags with controllable distance distributions. After fragmentation and ligation with common adaptors, genomic DNA is then subjected to one of the several protocols that results in an array of millions of spatially immobilized PCR colonies: this step can be achieved by several approaches, including *in situ* polonies, emulsion PCR or bridge PCR. Once the PCR colonies are immobilized in the array, the sequencing process itself consists of alternating cycles of enzyme-driven biochemistry and imaging-based data acquisition. The currently available HTS technologies include Illumina Genome Analyzer (GA), Applied Biosystem’s (ABI) SOLiD, Roche’s 454 and Helicos’ Heliscope sequencing machines (Table 1).

### 2.1. Roche 454 GenomeSequencer

The GenomeSequencer instrument was introduced in 2005 as the first next-generation system on the market by 454 Life Sciences. The basis of the 454 GenomeSequencer is the pyrophosphate detection that was first described in 1985 by Nyren Et, al. [9] and a system using this principle in a new method for DNA sequencing was reported in 1988 by Hyman Et, al. [10]. In this sequencing system, DNA fragments are ligated to beads by means of specific adapters. To obtain sufficient light signal intensity for detection in the sequencing-by-synthesis reaction step, emulsion PCR is carried out for amplification. Once the PCR amplification cycles are complete, each bead with its fragment is placed at the top end of an optical fiber that has the other end facing to a sensitive CCD camera, which enables the positional detection of emitted light. In the last step, polymerase enzyme and primer are added to the beads so that the synthesis of the complementary strand can start: the incorporation of a base by the polymerase enzyme in the growing chain releases a pyrophosphate group, which can be detected as emitted light.

A limitation of the 454 sequencing platform is that base calling cannot properly interpret long stretches (>6) of the same nucleotide (homopolymer DNA segments); for this reason homopolymer segments are prone to base insertion and deletion errors during base calling. By contrast, substitution errors are rarely encountered in Roche/454 sequence reads. Average raw error-rates are on the order of 0.1% [7]. At present, the GS FLX Titanium series allows generation of more than 1,000,000 single reads per run with an average read length of 400 bases. The device, schema of operation, its further developments and a list of publications with applications can be found on the 454 website [11]. 

### 2.2. Illumina Genome Analyzer

The Illumina Genome Analyzer (also called Solexa sequencer) has its origins in work by Turcatti and colleagues [12,13] and is the most widely available HTS technology. In this platform, the amplified sequencing features are generated by bridge PCR [12,14] and after immobilization in the array, all the molecules are sequenced in parallel by means of sequencing by synthesis. 

During the sequencing process, each nucleotide is recorded through imaging techniques, and is then converted into base calls. The Illumina sequencer is able to sequence reads up to 100 bp (with longer ones expected in the near future) with relatively low error rates. Read-lengths are limited by multiple factors that cause signal decay and dephasing, such as incomplete cleavage of fluorescent labels or terminating moieties. The great majority of the sequencing errors are substitution errors, while insertion/deletion errors are much less common. Average raw error-rates are on the order of 1–1.5% [15], but higher accuracy bases with error rates of 0.1% or less can be identified through quality metrics associated with each base-call. 

The latest Illumina Genome Analyzer IIe is able to generate up to 200 million 100 bp paired-end reads per run for a total of 20 Gb of data with a throughput of around 2 Gb per day. Information about the Genome Analyzer system can be found on the Solexa website [15].

### 2.3. ABI's SOLiD

The ABI SOLiD sequencer is another widely used sequencing platform and has its origins in the system described by Shendure *et al.* [16] in 2005 and in work by McKernan *et al.* [17] at Agencourt Personal Genomics (Beverly, MA, USA) (acquired by Applied Biosystems (Foster City, CA, USA) in 2006). The sequencing process used by ABI SOLiD is very similar to the Solexa work flow, however, there are also some differences. First of all, the clonal sequencing features are generated by emulsion PCR, instead of bridge PCR. Second, the SOLiD system uses a di-base sequencing technique in which two nucleotides are read (via sequencing by ligation) simultaneously at every step of the sequencing process, while the Illumina system reads the DNA sequences directly. Although there are 16 possible pairs of di-bases, the SOLiD system uses only four dyes and so sets of four di-bases are all represented by a single color. As the sequencing machine moves along the read, each base is interrogated twice: first as the right nucleotide of a pair, and then as the left one. In this way, it is possible to derive each subsequent letter if we know the previous one, and if one of the colors in a read is misidentified (e.g. due to a sequencing error), this will change all of the subsequent letters in the translation. Even if this may seem to generate problems in read sequencing, it can be advantageous during the read alignment to a reference genome. The raw ‘per-color’ error rate is around 2-4% [18]. The latest ABI SOLiD 4 machines are able to generate up to 1 billion 50 bp paired-end reads per run for a total of 100 Gb of data with a throughput of around 5 Gb per day. For further information see the Applied Biosystems website [18].

### 2.4. Single Molecule Sequencing

The origins of the Single Molecule Sequencing (SMS) date back to the work of Jett *et al.* [19], and the Heliscope sequencer, sold by Helicos, is the first commercial product that allows for sequencing with this technology. The Heliscope sequencer is based on cyclic interrogation of a dense array of sequencing features, but the unique aspect of this platform is that no clonal amplification is required. , A highly sensitive fluorescence detection system is used for the interrogation of single DNA molecules via sequencing by synthesis. At present, the error distribution of SMS technologies is much higher than that of PCR-based methods: this is due to the fact that since one physical piece of DNA is sequenced at a time, the sequencing signal is much weaker, leading to a large number of ‘dark bases’. The dominant error type is deletions (2–7% error rate with one pass; 0.2–1% with two passes). However, substitution error rates are substantially lower (0.01–1% with one pass). The latest Helicos Genetic Analysis System is able to generate up to 1 billion 35 bp reads per run for a total of 35 Gb of data [20].

There has been relatively little work toward developing informatics solutions for SMS data, and this is a very promising field for future algorithm development, as large SMS data sets are becoming available [21].

**Table 1:** Summary of the main features of the four HTS technologies.

	Roche 454	Illumina Genome Analyzer	ABI SOLiD	Helicos Heliscope
Sequencing method	Pyrosequencing	Reversible dye terminators	Sequencing by ligation	Single Molecule Sequencing
Read lengths	400 bases	100 bases	50 bases	35 bases
Sequencing run time	10 h	10 days	11-12 days	30 days
Total bases per run	500 Mb	20 Gb	100 Gb	35 Gb
Error Rate	0.1%	1.5%	4%	2-7%

### 2.5. Paired-end and mate-pair sequencing

All the sequencing technologies introduced above are able to generate paired-end or mate-pair data. Mate-pairs are created when genomic DNA is fragmented and size-selected inserts are circularized and linked by means of an internal adaptor. After purification, the mate-pairs are generated by sequencing around the adaptor. Paired-end reads, by contrast, are generated by the fragmentation of genomic DNA into short (<300 bp) segments, followed by sequencing of both ends of the segment. Although the approaches to obtain mate-pair and pair-end libraries are very different, from a computational perspective the distinction between mate-pairs and paired-ends is not crucial: paired reads are two sequences, generated at an approximately known distance from each other in the genome (the insert size). Paired reads are very useful for short-read data analysis: during the alignment process, a large fraction of short reads are difficult to map uniquely to the genome, and the second read of a pair can be used to find the correct location. Moreover, as we will see in the next chapters, mate-pairs are also typically used to discover structural variants (SVs)—regions of the genome that have undergone large-scale mutations, such as inversions and large insertions and deletions. 

## 3. Methods for alignment, assembly and polymorphism detection

### 3.1. Alignment

The first important challenge presented by HTS technologies data is the so-called read alignment (or mapping) problem. All the HTS platforms in production are able to produce data of the order of giga base-pairs (Gbp) per machine day [22]. With the emergence of such data, researchers have realized that traditional tools for aligning capillary reads are not efficient for this huge amount of data. For this reason, many new alignment tools have been developed in the last two years. These new tools use the many advantages specific to each of the new sequencing technologies, such as the short sequence length of Solexa, SOLiD and Helicos reads, the low indel error rate of Illumina reads and the di-base encoding of SOLiD reads. These new tools, named Short read aligners, outperform the performance of traditional aligners (such as BLAST [23]) in terms of both speed and accuracy. An algorithm for the alignment of short sequence reads produced by HTS technologies must be able to i) quickly and efficiently align the billions of short reads produced by this technique and ii) permit the alignment of non-unique reads (repetitive element in the reference) and of reads that do not match exactly the reference genome (sequencing errors or variations). 

In the last two years, more than 20 short-read alignment softwares have been published. A selection of freely available short read alignment softwares is reported in Table 2. 

All of the short reads alignment tools listed in Table 2 are able to output alignments in the SAM format [29], the emerging standard alignment format which is widely supported by alignment viewers. BWA and Mosaik work well for Sanger and 454 reads, allowing gaps and clipping. Bowtie and MAQ allow base quality scores to be used, improving alignment accuracy. MAQ only does gapped alignment for Illumina paired-end reads. All of the tools reported in Table 2 allow use of paired-end mapping. Paired-end alignment outperforms single-end alignment in terms of both sensitivity and specificity, allowing for a smaller number of wrongly mapped reads [30]. On speed, Bowtie, BWA and SOAP2 align ~7 Gbp against the human genome per CPU day, outperforming the other short read aligners.

**Table 2:** A selection of short reads alignment tools. The platform compatibility depends on the maximum read length supported by the program.

Program	Author(s)	Website	Platform	Aligned Gbp per CPU day
Maq [24]	Li H	http://maq.sourceforge.net/	Illumina, SOLiD (partial)	~ 0.2
Bowtie [25]	Langmead B Et, al.	http://bowtie-bio.sourceforge.net/index.shtml	Illumina	~ 7
SSAHA2 [26]	Ning Z Et, al.	http://www.sanger.ac.uk/resources/software/ssaha2/	Illumina, SOLiD, 454	~ 0.5
BWA [27]	Li H and Durbin R	http://bio-bwa.sourceforge.net/bwa.shtml	Illumina, SOLiD, 454	~ 7
SOAP2 [28]	Li R Et, al.	http://www.sanger.ac.uk/resources/software/ssaha2/	Illumina	~ 7

### 3.2. De novo Assembly

Tools that allow for the *de novo* short read assembly are essential when a reference genome does not exist or, in general, when a novel genome assembly is desired. In the last two years, many algorithms have been proposed for the *de novo* assembly, especially for bacterial genomes, including AbySS [31], ALLPATHS [32], Edena [33], Velvet [34] and SOAPdenovo [35]. All these programs are based on the de Bruijn graph data structure [36,37] and differ in how they treat errors and if they use read-pair information. To date, *de novo* assembly of the human genome from HTS data is able only to reconstruct short DNA regions (contigs), as the presence of repeats makes it difficult or impossible to assemble longer pieces. 

### 3.3. SNP / indel detection

SNP and inversion-deletion (indel) identification is a very important task when one deals with re-sequenced genomes. However, only an handful of tools have been implemented [24,26,35,38] for SNP and small (1–5 bp) indel discovery. The goal of these programs consist in judging the likelihood that a locus is a heterozygous or homozygous variant given the error rates of the platform, the probability of bad mappings, and the amount of coverage. For these reasons, all the available tools for SNP and indel discovery follow two main steps: the first is for data preparation and in the second each nucleotide is called under a bayesian framework.

In the first step (preparation step) each read is evaluated and filtered. Reads that may map to paralogs or repeat sequences are discarder or considered only if other reads give supporting evidence, quality values are reassigned based on various statistics and lastly a re-alignment step is employed to better align small indels.

After the preparation step a bayesian approach is applied to the filtered data. This approach consists of computing the conditional likelihood of the nucleotides at each position by using the Bayes rule:

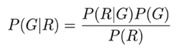
(1)

The Bayes rule states that the posterior probability P(G|R) of a certain genotype G given the data R can be calculated knowing the prior probability of that genotype and the probability of observing the given data from this genotype P(R|G) (likelihood). Usually, the prior P(G) is calculated as the probability of the variant while the probability of observing the prepared reads P(R|G) is then estimated for each possible donor genotype. The tools that use a Bayesian approach are PolyBayes [38], SOAPsnp [28] and MAQ [24]. 

Recently, two alternative methods to the Bayesian approach have been proposed by Malhis *et al*. [39] and by Hoberman *et al.* [40].

The algorithm proposed by Malhis *et al.* [39] is implemented in the Slider tool [41] and takes into consideration not just the most likely base at every position of a read, but also other possible bases. If there is a match between the most likely base and the reference allele, the match is considered nonvariant. If the most likely base does not match with the reference allele, but is above a cut-off probability, the base is considered variable, while if the reference allele is unlikely, the base is inferred as a candidate SNP.

Hoberman *et al.* [40] proposed an SNP detection algorithm based on a machine learning approach. Site-specific features are generated from read mappings, and this information is used to train a classifier. This classifier is then used to score the heterozygosity at each position.

### 3.4. Alignment / Assembly Viewers

**Figure 1:**
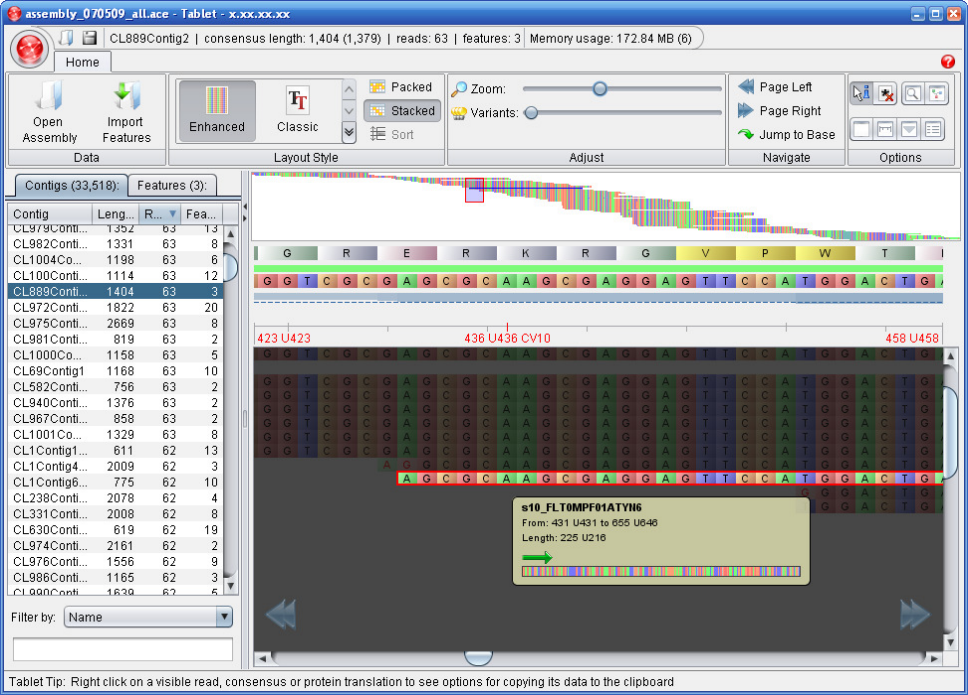
A screenshot of Tablet, an Alignment / Assembly visualization software. Figure taken from [60].

The advent of HTS technologies has brought about a need for fast, efﬁcient and user-friendly tools for browsing the resultant assemblies or alignments and the re-sequenced genomes. Tools that allow for the visualization of the alignment or assembly of short read data include EagleView [42], MapView [43], the Text Alignment Viewer of SAMtools [29], MaqView [24], Tablet [44] and IGV [45] by Broad Institute (Table 3 and Figure 1). 

When dealing with NGS data, visualization software is required that takes into account the following challenges: processing quickly and efficiently a huge amount of reads, providing high-quality rendering and navigation of the assembled reads and supporting a widening range of assembly formats. Moreover, the increasing diffusion of NGS technologies needs for biologist-friendly softwares with a user-friendly interface and for a range of common platforms. 

## 4. Methods for the detection of Structural Variants

The discovery of Structural Variants (SVs) is deeply changing our understanding of the human genotype. In the last decade, SVs detection has been performed with microarray technologies. The high-density CGH arrays (aCGH) and SNP genotyping arrays afford a level of resolution that allows CNV boundaries to be called with relatively high precision at a genome-wide level. However, although microarray platforms have been successfully used to identify CNVs [46,47,48], their resolution is limited by either the density of the array itself (for aCGH) or by the density of known SNP loci (for SNP arrays). For instance, currently available array platforms that consist of more than one million probes have a lower limit of detection of ~10−25 Kb [49,50]. The advent of HTS platforms has opened many opportunities for SV discovery and has enabled initiatives such as the 1000 Genomes project [51] that aims to sequence the genomes of more than 1000 individuals to extend our knowledge on human genetic variation. 

**Table 3:** A list of tools for the visualization of alignments or assemblies of short read data.

Program	Author(s)	Website	Distribution
EagleView	Huang W and Marth G	http://bioinformatics.bc.edu/marthlab/EagleView	Binary version for Windows, Mac OS X and Linux
MapView	Bao H Et, al.	http://202.116.74.148/mapview/l	Binary version for Windows and Linux
MaqView	Li H Et, al.	http://maq.sourceforge.net/maqview.shtml	Source Code (C, Java) and Binary version for Linux and Mac OS X
Tablet	Milne I Et, al.	http://bioinf.scri.ac.uk/tablet/	Binary version for Windows, Mac OS X and Linux
IGV	Broad Institute	http://www.broadinstitute.org/igv	Binary version for Windows, Mac OS X and Linux

The ﬁrst HTS-based approach to detect SVs were based on paired-end read mapping (PEM), which identiﬁes insertions and deletions by comparing the distance between mapped read pairs to the average insert size of the genomic library. Although this method is able to identify deletions smaller than 1 Kb with high sensitivity, it does not allow the discovery of insertions larger than the average insert size of the library and the exact borders of SVs in complex genomic regions rich in segmental duplication [52]. In this scenario, a very promising approach for the identiﬁcation of SVs using HTS technologies consists in measuring the depth of coverage (DOC) of reads aligned to the human reference genome. At present, few computational methods have been developed for the analysis of DOC data: Campbell *et al.* [8] use the Circular Binary Segmentation algorithm [53] originally developed for genomic hybridization microarray data, Chiang *et al.* [6] use a local change-point analysis technique, Yoon *et al.* [54] developed a new statistical method based on signiﬁcance testing that works on intervals of data points, while Magi *et al.* [55] developed a novel algorithm, named JointSLM, that allows them to analyze DOC signals from multiple samples simultaneously.

### 4.1. PEM-based Methods

Pair-end sequencing means to sequence both ends of a DNA fragment. In this way, the two reads belonging to a pair will have a certain distance on the genome (mapped distance). The mapped distance is compared to the expected insert size: in case that the mate-pair overlaps a SV, the distance and the orientations will be different in comparison to the expected insert size. When an insertion (deletion) occurs, the mapped distance will be smaller (larger) than the expected insert size. When an inversion occurs, the orientation of one of the two mappings will be opposite from the expected. However, a single mate-pair is not sufficient to predict an SV [52] and a clustering step is required to support each putative event. Several PEM-based algorithms have been developed for the detection of SVs, including PEMer [56], VariationHunter [57], MoDIL [58] and BreakDancer [59]. These tools mainly differ on the variant of signatures they detect and on the clustering procedures (Table 4). 

### 4.2. DOC-based Methods

The use of PEM-based methods does not allow for the discovery of all types of SVs [52]. An alternate approach for the detection of SVs is by analyzing the read depth of coverage (DOC) signal. The copy number of any genomic region can be estimated by counting the number of aligned reads to the reference genome. The strategy to obtain DOC data consists of counting the number of mapped reads in non-overlapping windows of ﬁxed length and then correcting each window by GC content [54]. 

The DOC data obtained with this approach is mathematically very similar to the signal obtained from aCGH log_2_-ratios. Deletions or duplications are identiﬁed as a decrease or increase in coverage across multiple consecutive windows. Moreover, like aCGH log_2_-ratios., DOC sequences have noise caused by mapping errors and random ﬂuctuations in genome coverage. For these reasons, the events in DOC can be detected using the same types of segmentation algorithms that are used for aCGH data. Campbell *et al.* [8] and Chiang *et al.* [6] were the ﬁrst to use this approach to detect copy-number alterations between tumor and healthy samples of the same individuals, while more recently Yoon *et al.* [54] proposed to use the read count in sequence data to look for genomic regions that differ in copy number between normal individuals of the 1000 genomes project. 

A very useful tool for the preparation of the GC-normalized DOC data is RDXplorer [61], which estimates the coverage of RD in 100-bp non-overlapping windows across an individual genome. Moreover, RDXplorer allows for the detection of SVs in multiple genomes by using an Event-Wise Testing (EWT) algorithm [54]. RDXplorer accepts the Sequence Alignment/Map (SAM) binary (BAM) file format as input and generates ready to use CNV call sets. 

**Table 4:** A selection of PEM-based algorithms for the detection of Structural Variants.

Program	Author(s)	Website	Detectable Events
PEMer	Korbel J Et, al.	http://sv.gersteinlab.org/pemer/	basic deletion, basic insertion, basic inversion, linking, linked insertion
VariationHunter	Hormozdiari F Et, al.	http://compbio.cs.sfu.ca/strvar.htm	basic deletion, basic insertion, basic inversion, everted duplication
MoDIL	Lee S Et, al.	http://compbio.cs.toronto.edu/modil/	basic deletion, basic insertion
BreakDancer	Chen K Et, al.	The software package is available as supplementary information at Nature Methods Online	basic deletion, basic insertion, basic inversion, hanging insertion

## 5. Conclusions

The emergence of High Throughput Sequencing technologies is enabling sequencing of genomes at a significantly lower cost, while opening a new scenario in our knowledge of the human genotype.

To date, a variety of software tools are available for analyzing next-generation sequencing data, ranging from short-read alignment programs to algorithms for the detection of structural variants. A comprehensive list of relevant software can be found on the SEQanswers website [62].

However, although all the sections discussed in this review describe the tremendous progress achieved over the last several years in analyzing HTS data, much work remains. First, algorithms for the analysis of the DOC data should be improved in order to obtain higher resolution in the identification of structural variants smaller than 1 Kb. At present, this task has been faced by using segmentation algorithms already developed for array-CGH data. Second, even if several assembly tools have been adapted or developed for the reconstruction of full human genotypes from short reads, this task remains an extremely challenging problem. However, HTS technologies based on SMS promise to increase read length to thousands of base pairs [63] allowing for the improvement of the performance of the assembly algorithms. Finally, there is the need for novel algorithms that allow data from different platforms to be combined in order to have a major impact on the overall success of *de novo* assembly [64,65].

In light of the ability to accurately and systematically determine the absolute copy number for any genomic segment, we anticipate that HTS technologies will eventually replace aCGH–based platforms for the discovery of new structural variants.

As these sequencing platforms becomes more commonplace, there is an increasingly need for data specialist to extract biological information from the huge amounts of data produced. Therefore, a key task is to get a clear picture of the bioinformatic tools available for the NGS data analysis.

## References

[1] Mitra R.D., Church  G.M. (1999). *In situ* localized amplification and contact replication of many individual DNA molecules. Nucleic Acids Res..

[2] Nagalakshmi U., Wang Z., Waern K., Shou C., Raha D., Gerstein M., Snyder M. (2008). The transcriptional landscape of the yeast genome defined by RNA sequencing. Science.

[3] Mortazavi A., Williams B.A., McCue K., Schaeffer L., Wold B. (2008). Mapping and quantifying mammalian transcriptomes by RNA-Seq. Nat. Methods.

[4] Wang Z., Gerstein M., Snyder M. (2009). RNA-Seq: a revolutionary tool for transcriptomics. Nat. Rev. Genet..

[5] Park P.J. (2009). ChIP-seq: advantages and challenges of a maturing technology. Nat. Rev. Genet..

[6] Chiang D.Y., Getz G, Jaffe D.B., O’Kelly M.J.T., Zhao X, Carter S.L., Russ C., Nusbaum C., Meyerson M., Lander E.S. (2009). High-resolution mapping of copy-number alterations with massively parallel sequencing. Nat. Methods.

[7] Alkan C., Kidd J.M., Marques-Bonet T., Aksay G., Antonacci F., Hormozdiari F., Kitzman J.O., Baker C., Malig M., Mutlu O. (2009). Personalized copy number and segmental duplication maps using next-generation sequencing. Nat. Genet..

[8] Campbell P.J., Stephens P.J., Pleasance E.D., O’Meara S., Li H., Santarius T., Stebbings L.A., Leroy C., Edkins S. (2008). Identification of somatically acquired rearrangements in cancer using genome-wide massively parallel paired-end sequencing. Nat. Genet..

[9] Nyren P., Lundin A. (1985). Enzymatic method for continuous monitoring of inorganic pyrophosphate synthesis. Anal. Biochem..

[10] Hyman E.D. (1988). A new method of sequencing DNA. Anal. Biochem..

[11] 454 Home Page. http://www.454.com/indecx.asp.

[12] Fedurco M., Romieu A., Williams S., Lawrence I., Turcatti G.  (2006). BTA, a novel reagent for DNA attachment on glass and efficient generation of solid-phase amplified DNA colonies. Nucleic Acids Res..

[13] Turcatti G., Romieu A., Fedurco M., Tairi A.P. (2008). A new class of cleavable fluorescent nucleotides: synthesis and 
optimization as reversible terminators for DNA sequencing by synthesis. Nucleic Acids Res..

[14] Adessi C., Matton G., Ayala G., Turcatti G., Mermod J.J., Mayer P., Kawashima E. (2000). Solid phase DNA amplification: characterisation of primer attachment and amplification mechanisms. Nucleic Acids Res..

[15] Solexa Home Page. http://www.solexa.com/.

[16] Shendure J., Porreca G.J., Reppas N.B., Lin X., McCutcheon J.P., Rosenbaum A.M., Wang M.D., Zhang K., Mitra R.D., Church G.M. (2005). Accurate multiplex polony sequencing of an evolved bacterial genome. Science.

[17] McKernan K., Blanchard A., Kotler L., Costa G. (2006). Reagents, methods, and libraries for bead-based sequencing. US patent application.

[18] Applied Biosystems Home Page. www3.appliedbiosystems.com/index.htm.

[19] Jett J.H., Keller R.A., Martin J.C., Marrone B.L., Moyzis R.K., Ratliff R.L., Seitzinger N.K., Shera E.B., Stewart C.C. (1989). High-speed DNA sequencing: an approach based upon fluorescence detection of single molecules. J. Biomol. Struct. Dyn..

[20] Helicos Home Page. http://www.helicosbio.com/.

[21] Pushkarev D., Neff N.F., Quake S.R. (2009). Single-molecule sequencing of an individual human genome. Nat. Biotechnol..

[22] Metzker M.L. (2010). Sequencing technologies – the next generation. Nat. Rev. Genet..

[23] Kent W.J. (2002). BLAT--the BLAST-like alignment tool. Genome Res..

[24] Li H., Ruan J., Durbin R. (2008). Mapping short DNA sequencing reads and calling variants using mapping quality scores. Genome Res..

[25] Langmead B., Trapnell C., Pop M., Salzberg S.L. (2009). Ultrafast and memory-efficient alignment of short DNA sequences to the human genome. Genome Biol..

[26] Ning Z., Cox A.J., Mullikin J.C. (2001). SSAHA: a fast search method for large DNA databases. Genome Res..

[27] Li H., Durbin R. (2010). Fast and accurate long-read alignment with Burrows-Wheeler transform. Bioinformatics.

[28] Li R., Yu C., Li Y., Lam T., Yiu S., Kristiansen K., Wang J. (2009). SOAP2: an improved ultrafast tool for short read alignment. Bioinformatics.

[29] Li H., Handsaker B., Wysoker A., Fennell T., Ruan J., Homer N., Marth G., Abecasis G., Durbin R. (2009). The Sequence Alignment/Map format and SAMtools. Bioinformatics.

[30] Li H., Homer N. (2010). A survey of sequence alignment algorithms for next-generation sequencing. Brief. Bioinform..

[31] Simpson J.T., Wong K., Jackman S.D., Schein J.E., Jones S.J.M., Birol I. (2009). ABySS: a parallel assembler for short read sequence data. Genome Res..

[32] Butler J., MacCallum I., Kleber M., Shlyakhter I.A., Belmonte M.K., Lander E.S., Nusbaum C., Jaffe D.B. (2008). ALLPATHS: *de novo* assembly of whole-genome shotgun microreads. Genome Res..

[33] Hernandez D., François P., Farinelli L., Osteras M., Schrenzel J. (2008). De novo bacterial genome sequencing: millions of very short reads assembled on a desktop computer. Genome Res..

[34] Zerbino D.R., Birney E. (2008). Velvet: algorithms for *de novo* short read assembly using de Bruijn graphs. Genome. Res..

[35] Li R., Li Y., Kristiansen K., Wang J. (2008). SOAP: short oligonucleotide alignment program. Bioinformatics.

[36] Pevzner P.A., Borodovsky M.Y., Mironov A.A. (1989). Linguistics of nucleotide sequences. II: Stationary words in genetic texts and the zonal structure of DNA. J. Biomol. Struct. Dyn..

[37] Idury R.M., Waterman M.S. (1995). A new algorithm for DNA sequence assembly. J. Comput. Biol..

[38] Marth G.T., Korf I., Yandell M.D., Yeh R.T., Gu Z., Zakeri H., Stitziel N.O., Hillier L., Kwok P.Y., Gish W.R. (1999). A general approach to single-nucleotide polymorphism discovery. Nat. Genet..

[39] Malhis N., Jones S.J.M. (2010). High quality SNP calling using Illumina data at shallow coverage. Bioinformatics.

[40] Hoberman R., Dias J., Ge B., Harmsen E., Mayhew M., Verlaan D.J., Kwan T., Dewar K., Blanchette M., Pastinen T. (2009). A probabilistic approach for SNP discovery in high-throughput human resequencing data. Genome Res..

[41] Malhis N., Butterfield Y.S., Ester M., Jones S.J. (2009). Slider--maximum use of probability information for alignment of short sequence reads and SNP detection. Bioinformatics.

[42] Huang W., Marth G. (2008). EagleView: a genome assembly viewer for next-generation sequencing technologies. Genome Res..

[43] Bao H., Guo H., Wang J., Zhou R., Lu X., Shi S. (2009). MapView: visualization of short reads alignment on a desktop computer. Bioinformatics.

[44] Milne I., Bayer M., Cardle L., Shaw P., Stephen G., Wright F., Marshall D. (2010). Tablet—next generation sequence assembly visualization. Bioinformatics.

[45] IGV Software Home Page. http://www.broadinstitute.org/igv.

[46] Iafrate A.J., Feuk L., Rivera M.N., Listewnik M.L., Donahoe P.K., Qi Y., Scherer S.W., Lee C. (2004). Detection of large-scale variation in the human genome. Nat. Genet..

[47] Redon R., Ishikawa S., Fitch K.R., Feuk L., Perry G.H., Andrews T.D., Fiegler H., Shapero M.H., Carson A.R., Wenwei Chen W. (2006). Global variation in copy number in the human genome. Nature.

[48] Conrad D.F., Pinto D., Redon R., Feuk L., Gokcumen O., Zhang Y., Aerts J., Andrews T.D., Barnes C., Campbell P. (2010). Origins and functional impact of copy number variation in the human genome. Nature.

[49] McCarroll S., Kuruvilla F., Korn J., Cawley S., Nemesh J., Wysoker A., Shapero M., de Bakker P., Maller J., Kirby A. (2008). Integrated detection and population-genetic analysis of SNPs and copy number variation. Nat. Genet..

[50] Cooper G.M., Zerr T., Kidd J.M., Eichler E.E., Nickerson D.A.  (2008). Systematic assessment of copy number variant detection via genome-wide SNP genotyping. Nat. Genet..

[51] 1000 Genomes Project Home Page. http://www.1000genomes.org.

[52] Dalca A.V., Brudno M. (2010). Genome variation discovery with high-throughput sequencing data. Brief. Bioinform..

[53] Olshen A.B., Venkatraman E.S., Lucito R., Wigler M. (2005). Circular binary segmentation for the analysis of array-based DNA copy number data. Biostatistics.

[54] Yoon S., Xuan Z., Makarov V., Ye K., Sebat J. (2009). Sensitive and accurate detection of copy number variants using read depth of coverage. Genome Res..

[55] Magi A., Benelli M., Seungtai Yoon S., Torricelli F.  Detecting Common Copy Number Variants in High-Throughput Sequencing Data by using JointSLM algorithm. Nucleic Acids Res..

[56] Korbel J.O., Abyzov A., Mu X.J., Carriero N., Cayting P., Zhang Z., Snyder M.;Gerstein (2009). PEMer: a computational framework with simulation-based error models for 
inferring genomic structural variants from massive paired-end 
sequencing data. Genome Biol..

[57] Hormozdiari F., Alkan C., Eichler E.E., Sahinalp S.C. (2009). Combinatorial algorithms for structural variation detection in high-throughput sequenced genomes. Genome Res..

[58] Lee S., Hormozdiari F., Alkan C., Brudno M. (2009). MoDIL: detecting small indels from clone-end sequencing with mixtures of distributions. Nat. Methods.

[59] Chen K., Wallis J.W., McLellan M.D., Larson D.E., Kalicki J.M., Pohl C.S., McGrath S.D., Wendl M.C., Zhang Q., Locke D.P. (2009). BreakDancer: an algorithm for high-resolution mapping of genomic structural variation. Nat. Methods.

[60] Tablet Home Page. http://bioinf.scri.ac.uk/tablet/index.shtml.

[61] Rdxplorer Home Page. http://rdxplorer.sourceforge.net/.

[62] Seqanswer Home Page. http://seqanswers.com/.

[63] Eid J., Fehr A., Gray J., Luong K., Lyle J., Otto G., Peluso P., Rank D., Baybayan P., Bettman B. (2009). Real-time DNA sequencing from single polymerase molecules. Science.

[64] Aury J., Cruaud C., Barbe V., Rogier O., Mangenot S., Samson G., Poulain J., Anthouard V., Scarpelli C., Artiguenave F. (2008). High quality draft sequences for prokaryotic genomes using a mix of new sequencing technologies. BMC Genomics.

[65] Reinhardt J.A., Baltrus D.A., Nishimura M.T., Jeck W.R., Jones C.D., Dangl J.L. (2009). De novo assembly using low-coverage short read sequence data from the rice pathogen Pseudomonas syringae pv. oryzae. Genome Res..

